# Impaired Retention of Motor Learning of Writing Skills in Patients with Parkinson’s Disease with Freezing of Gait

**DOI:** 10.1371/journal.pone.0148933

**Published:** 2016-02-10

**Authors:** Elke Heremans, Evelien Nackaerts, Griet Vervoort, Sanne Broeder, Stephan P. Swinnen, Alice Nieuwboer

**Affiliations:** 1 Neuromotor Rehabilitation Research Group—Department of Rehabilitation Sciences—KU, Leuven, Belgium; 2 Movement Control and Neuroplasticity Research Group—Department of Kinesiology—KU, Leuven, Belgium; Federal University of Rio de Janeiro, BRAZIL

## Abstract

**Background:**

Patients with Parkinson’s disease (PD) and freezing of gait (FOG) suffer from more impaired motor and cognitive functioning than their non-freezing counterparts. This underlies an even higher need for targeted rehabilitation programs in this group. However, so far it is unclear whether FOG affects the ability for consolidation and generalization of motor learning and thus the efficacy of rehabilitation.

**Objective:**

To investigate the hallmarks of motor learning in people with FOG compared to those without by comparing the effects of an intensive motor learning program to improve handwriting.

**Methods:**

Thirty five patients with PD, including 19 without and 16 with FOG received six weeks of handwriting training consisting of exercises provided on paper and on a touch-sensitive writing tablet. Writing training was based on single- and dual-task writing and was supported by means of visual target zones. To investigate automatization, generalization and retention of learning, writing performance was assessed before and after training in the presence and absence of cues and dual tasking and after a six-week retention period. Writing amplitude was measured as primary outcome measure and variability of writing and dual-task accuracy as secondary outcomes.

**Results:**

Significant learning effects were present on all outcome measures in both groups, both for writing under single- and dual-task conditions. However, the gains in writing amplitude were not retained after a retention period of six weeks without training in the patient group without FOG. Furthermore, patients with FOG were highly dependent on the visual target zones, reflecting reduced generalization of learning in this group.

**Conclusions:**

Although short-term learning effects were present in both groups, generalization and retention of motor learning were specifically impaired in patients with PD and FOG. The results of this study underscore the importance of individualized rehabilitation protocols.

## Introduction

Patients with Parkinson’s disease (PD) typically suffer from a wide range of motor and non-motor problems. One of the most severe motor symptoms is freezing of gait (FOG), which is defined as a “brief, episodic absence or marked reduction of forward progression of the feet despite the intention to walk” [1]. While FOG is typically known as a lower extremity phenomenon, recent studies provide mounting evidence that freezing is a possible expression of a wider disturbance of motor control [2]. In addition, motor problems such as impaired gait outside the freezing episodes and disturbances in other repetitive movement sequences may also occur [2–4]. Heremans et al. [5] showed, for example, that handwriting problems are more pronounced in patients with FOG (PD+FOG) than those without (PD-FOG). PD+FOG had a greater decrease in writing amplitude, generally referred to as micrographia, compared to PD-FOG, irrespective of disease severity.

As most motor problems, including micrographia, are only partially improved by dopaminergic medication, there is a need for targeted rehabilitation programs in patients with PD [6,7]. Knowledge on the ability that patients have to learn new motor skills is pivotal for planning these programs. Motor learning is defined as improved performance resulting from practice, characterized by a reduction in motor variability and a degree of automatization that persists over time [8]. Effective consolidation of motor learning results in long-term retention and generalization of what was learned towards related or similar tasks performed in a different context. Increased automatization can be observed when task performance increases even under distracting circumstances, such as during dual tasking. A recent study in PD patients without FOG showed positive effects of amplitude training of handwriting which generalized to dual tasking [9]. However, it can be expected that the ability for motor learning differs with respect to the disease profile. As FOG is associated with more severe motor as well as cognitive impairments, people with this symptom may thus have more difficulty with retention and generalization of learning. Cognitive impairments were found in freezers in several domains of executive functioning and have been shown to extend to difficulties with implicit motor learning [10–14]. Vandenbossche et al. [14] showed that learning of a serial reaction time task in PD+FOG was negatively affected by adding a dual task, suggesting an impairment with reaching automaticity in this group.

So far, only a few studies have reported motor learning interventions to improve handwriting in PD and all, except one, were limited to short-term effects on the trained task only [9,15,16]. In addition, previous studies did not take into account potential learning differences between people with and without FOG. Furthermore, it is unclear how motor learning programs should be designed to optimally address the needs of different groups of PD patients. In PD in general, compensatory strategies such as external cueing were shown to enhance patients’ motor learning [17–19]. However, especially impaired executive functioning, may be accompanied with difficulties in employing such compensatory strategies due to insufficient cognitive reserve. This would predict that patients with FOG may be less able to benefit from cueing [20]. In accordance to this hypothesis, PD+FOG were found to be more prone to develop cue dependency, hindering transfer of learning towards task performance in the absence of these cues [4,21]. In contrast, other studies found positive effects of cueing in PD irrespective of FOG [22,23].

To address these questions, in the current study the effects of an intensive motor learning program designed to improve handwriting performance in patients with PD with and without FOG were investigated. The training program was supported by visual target zones, as this was previously shown to optimize treatment effects in PD [15,16]. Learning effects of both patient groups were compared in the light of the different hallmarks of motor learning, including automatization, generalization and retention. To investigate automatization, handwriting amplitude was compared before and after training during single- and dual-task training as primary outcome of the study, as improvements on this parameter are directly related to increased writing legibility. Variability of writing amplitude and dual-task accuracy were included as secondary outcome measures. Generalization was investigated by comparing cued and uncued writing performance and retention by looking at the gains that were retained after a six-week follow up period. It was hypothesized that external cueing would differentially affect PD-FOG and PD+FOG. As well, retention of motor learning was expected to be compromised in patients with FOG given their increased motor and cognitive impairments.

## Methods

### Participants

Thirty five participants with PD were grouped into non-freezers (n = 19, 11 males) and freezers (n = 16, 13 males), according to item 3 of the new freezing of gait questionnaire (NFOG-Q) [24]. This sample size was based on a power analysis with β = 0.20, α = 0.05 and writing amplitude data derived from Tucha et al. [25] and was shown to be sufficient to detect changes in writing size between groups of 20%. The patients recruited in this study show a partial overlap with those included in studies by Heremans et al. [5] and Nackaerts et al. [9]. Freezers and non-freezers were matched a priori according to age and disease severity as measured by the Hoehn and Yahr (H&Y) scale [26]. Inclusion criteria consisted of (i) a diagnosis of PD according to the United Kingdom PD Society Brain Bank criteria [27]; (ii) Hoehn and Yahr (H&Y) stage I to III in the on-phase of the medication cycle; (iii) handwriting problems, reflected by a score of 1 or more on the handwriting item (2.7) of the Movement Disorder Society-sponsored revision of the Unified Parkinson’s Disease Rating Scale (MDS-UPDRS) part II [28]; and (iv) Mini-Mental State Examination (MMSE) ≥ 24 [29]. Exclusion criteria were: (i) visual impairments, including color blindness; (ii) upper limb medical problems which would impede handwriting; (iii) a history of depression or neurological diseases other than PD and (iv) deep brain stimulation. The study design and protocol were approved by the local Ethics Committee of the KU Leuven and were in accordance with The Code of Ethics of the World Medical Association (Declaration of Helsinki, 1967). After complete explanation of the study protocol, written informed consent was obtained from all participants prior to participation in the experiment.

### Experimental Procedure and Tasks

**Fig 1:**
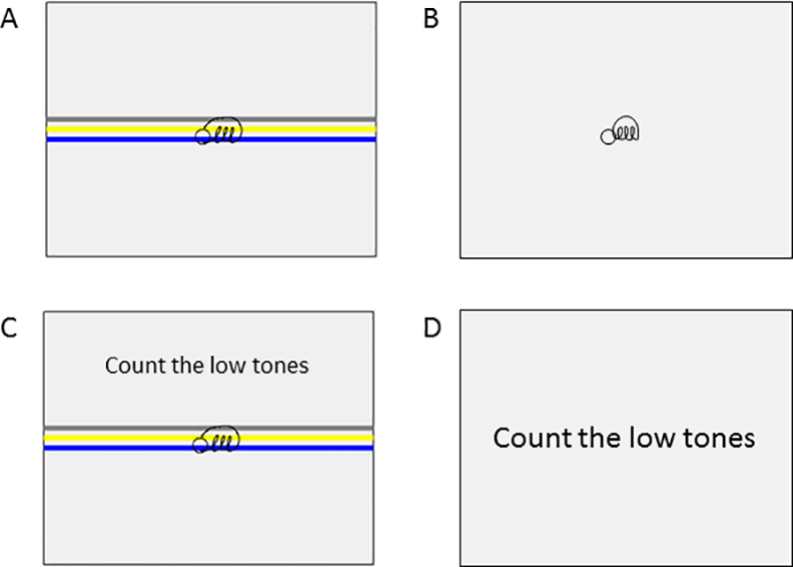
Writing tasks that were assessed by means of the touch-sensitive writing tablet. (A) Single-task writing with visual target zones, (B) Single-task writing without visual target zones, (C) Dual-task writing, (D) Single-task tone counting. The small circle indicates the starting point of the writing sequence and the colored target zones indicate the requested writing amplitude in the conditions with cues.

At the onset of the study, all participants completed a clinical test-battery including the (i) H&Y Scale, (ii) motor part of the MDS-UPDRS (part III), (iii) NFOG-Q and (iv) MMSE. Patients’ handwriting was tested before training, after six weeks of training and after a six-week retention period without training by means of a touch-sensitive writing tablet with a sampling frequency of 200Hz and spatial resolution of 32.5 μm [30,31]. All tests and training sessions were performed while patients were in the stable ‘on’-phase of the medication cycle, i.e. approximately 1 h after medication intake. Different writing tasks were used in order to investigate the three hallmarks of motor learning, i.e. generalization, automatization and retention. The transfer task, designed to investigate generalization of learning, consisted of writing continuous 3-loop sequences of repetitive cursive loops at either continuously small (0.6 cm) or large size (1 cm) in the presence and absence of visual target zones with a width of 2 mm. Instructions on the requested writing amplitude were displayed on the screen before each trial and each trial was initiated by a starting tone. In the condition without cues the visual target zones disappeared after 2 s, whereas in the condition with cues they remained present during the entire trial. Participants were instructed to start writing within the start circle and write three loops from the bottom of the blue target zone until the top of the yellow target zone and then return to the start circle by drawing a curved line through the upper gray line (Fig 1). When returning to the start circle, the previous loop disappeared from the screen, allowing continuous repetition of the 3-loop figure during the 27 s duration of the trial. Writing was performed at comfortable speed. All patients performed three blocks of four trials in which the writing conditions with and without cues and at small and large size were randomized.

Furthermore, all patients performed three blocks of four trials, consisting of single and dual-task writing in order to study movement automatization (automatization task) [30]. The single task was identical to the one described above. During dual tasking, the writing task had to be performed in combination with counting high- and low-pitched tones produced randomly at intervals of 3 s. Instructions on which tones to count were displayed on the screen before each trial. Participants were instructed to silently count either the high tones or the low tones and verbally report the total number to the examiner at the end of each trial. Task order was randomized across participants. Tests were performed in a quiet room while sitting at a table on a height-adjustable chair.

Between the first and second writing tests, all patients underwent intensive writing training during six weeks for 30 minutes a day, five times per week. Writing training was performed at home and consisted of a combination of paper-and-pencil exercises and exercises on the training tablet, aimed at improving writing size. The difficulty of the writing exercises was gradually increased over the course of the six weeks. During the first three weeks of practice, visual target zones with a thickness of 2 mm were provided as external cues to stimulate writing at large size. During the last three weeks, the thickness of the zones was decreased to 0.5 mm to increase the accuracy requirements. Over the six-week training period, progression was made from writing pre-letters to letters to words and from writing at one size (either 0.6 cm or 1 cm) to writing at alternating sizes. The 3-loop sequence that was used during the assessments was trained on a daily basis. Dual-task practice was added from the fourth week on and was provided in the same way as it was tested. During the training phase, all patients received weekly visits by one of the researchers to provide training instructions and feedback. Compliance of the participants was monitored by means of a diary.

### Data Processing

Kinematic data of the writing tablet were filtered at 7 Hz using a 4^th^ order Butterworth filter and analyzed with custom-written Matlab R2011b software (Mathworks) [30]. As a primary outcome, movement amplitude was determined by calculating the differences between the local minima and maxima of each stroke for each size separately and expressing this as a percentage of the requested writing size. As secondary outcome, the coefficient of variation of the within-patient writing amplitude (COV_ampl_), that is, the ratio of standard deviation to the mean expressed as a percentage, was computed. Furthermore, accuracy of the tone-counting task was determined as a percentage of correct answers.

### Statistical Analyses

Data were analyzed using SPSS software (version 22). Normality and equality of variance were checked for all variables. Demographic characteristics were compared between groups by means of independent t-tests or Mann-Whitney U tests depending on the distribution of the data. Retention data of one patient from the PD+FOG group were missing, as this patient experienced a stroke during the follow-up period and was not able to perform the retention test. The transfer task was analyzed by means of a linear mixed model with Group (PD-FOG, PD+FOG), Time (pre, post, retention), Size (small, large) and Cue (with cue, without cue) as fixed factors. For the automatization task, the data of one participant in each group had to be removed as they experienced hearing problems making it impossible to correctly perform the dual-task. A linear mixed model with Group, Time, Size and Task (single, dual) as fixed factors was used for the analysis of amplitude and COV_ampl_ in this task. Accuracy on the secondary task was analyzed by means of a linear mixed model with Group, Time and Task as fixed factors. For all analyses, MDS-UPDRS-III was included as a covariate as this variable differed significantly between patient groups. All models controlled for the within-subject differences by including random effects for participants. For all analyses, alpha was set at 0.05 and post hoc analyses were carried out using Bonferroni tests.

## Results

### Subjects

No significant differences between patients with and without FOG were found for age, gender, L-dopa equivalent dose (LED) intake, H&Y scale and MMSE-scores. A significant difference between PD-FOG and PD+FOG was found for MDS-UPDRS-III scores (p = 0.04) (Table 1). Significant differences in NFOG-Q scores (p<0.01) confirmed the validity of the subgroups. Compliance to the training was very high in both groups. PD-FOG completed 95.8% of their training sessions and PD+FOG 93.8%.

**Table 1:** Clinical characteristics of patients with Parkinson with and without FOG.

Parameter	PD-FOG	PD+FOG	*P*-value
Age (years)	63.4 (8.9)	64.7 (8.6)	0.65
Edinburgh Handedness Inventory	100 (87.5,100)	95 (40,100)	0.24
Disease duration (years)	7.3 (5.0)	8.8 (4.7)	0.37
MMSE (0–30)	29 (29,30)	28 (28,29)	0.39
H&Y (0-V)	2 (2,2)	2 (2,2)	0.95
LED (mg/24h)	571 (313)	560 (327)	0.92
MDS-UPDRS-III (0–108)	27.4 (12.1)	38.2 (17.5)	0.03
NFOG-Q (0–23)	0 (0,0)	14.1 (7.7)	<0.01

Abbreviations: MMSE = Mini Mental State Examination; H&Y = Hoehn and Yahr stage

LED = levodopa equivalent dose; MDS-UPDRS-III = Movement Disorders Unified

Parkinson’s disease rating scale part 3; NFOG-Q = New freezing of gait questionnaire.

Results are presented as mean and 1 standard deviation for normally distributed data and as the median and interquartile range (Q1,Q3) for non-normally distributed data.

### Movement Amplitude

**Fig 2:**
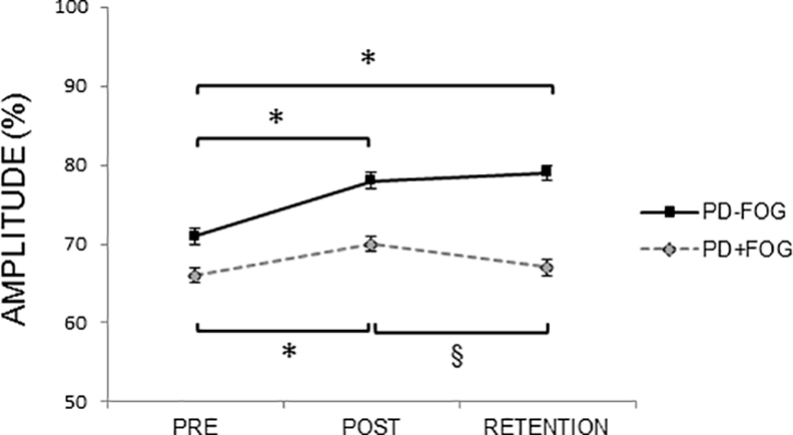
Writing amplitude on the transfer task during the pre, post and retention test for both groups. Mean and standard errors are presented. For writing amplitude (% of target size) a significant Time x Group interaction was found (F = 4.74, p = 0.01), showing improvements in amplitude from pre to post test in both groups, and from pre to retention test in the PD-FOG only. In the PD+FOG a significant decrease in amplitude was shown from post to retention test. * indicates significant increase with p<0.05, § indicates significant decrease with p<0.05.

**Fig 3:**
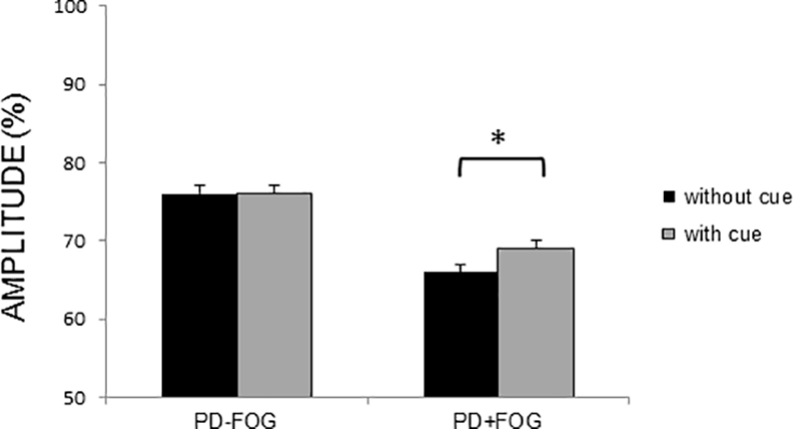
Writing amplitude on the transfer task in the presence and absence of cues for both groups. Mean and standard errors are presented. For writing amplitude (% of target size) a significant Group x Cue interaction was found (F = 4.42, p = 0.04), showing improved performance in the presence of cues in the PD+FOG only. * indicates significant increase with p<0.05.

For the transfer task (cued vs uncued writing), a significant interaction was found between Group and Time (F = 4.74, p = 0.01) (Fig 2). Writing amplitude did not differ between both groups at baseline, but significantly differed at post (p = 0.03) and retention tests (p<0.01). Post hoc tests showed an increased amplitude from baseline to post-training in both groups (PD-FOG: p<0.01; PD+FOG: p = 0.04). In the PD-FOG, significant differences remained between the pre and retention tests (p<0.01), which was not the case for the PD+FOG as the amplitude significantly decreased again from the post to the retention test (p<0.01) in this group. In addition, a significant interaction was found between Group and Cue (F = 4.42, p = 0.04) (Fig 3). Post hoc tests showed that there were no significant differences in amplitude between writing with and without cues in the PD-FOG, whereas in the PD+FOG writing size was significantly smaller during writing without cues in all conditions (p = 0.01), showing an increased cue dependency in this group. The significant interaction between Cue and Size (F = 43.14, p<0.01) indicated that writing size, expressed as a percentage of the requested size, was significantly larger in patients from both groups when they were requested to write at large size in comparison to small size and this both during writing with and without cues (p<0.01 for all comparisons).

For the automatization task (single- vs dual-task writing) a significant Time by Group interaction was found (F = 4.04, p = 0.02). Similar to what was observed for the transfer task, PD-FOG exhibited a significant increase in amplitude from pre to post test and from pre to retention test (p<0.01 for both comparisons). In contrast, the PD+FOG significantly increased their amplitude from pre to post test (p = 0.04), but the difference did not remain significant from the pre to retention test. In addition, for the automatization task a significant Size by Time interaction was found (F = 7.05, p<0.01). Post hoc tests showed that increases in amplitude from pre to post and pre to retention tests were only significant for writing at large size (p<0.01 for both comparisons), and did not reach significance for writing at small size.

### Coefficient of Variation of the Within-Patient Writing Amplitude

**Fig 4:**
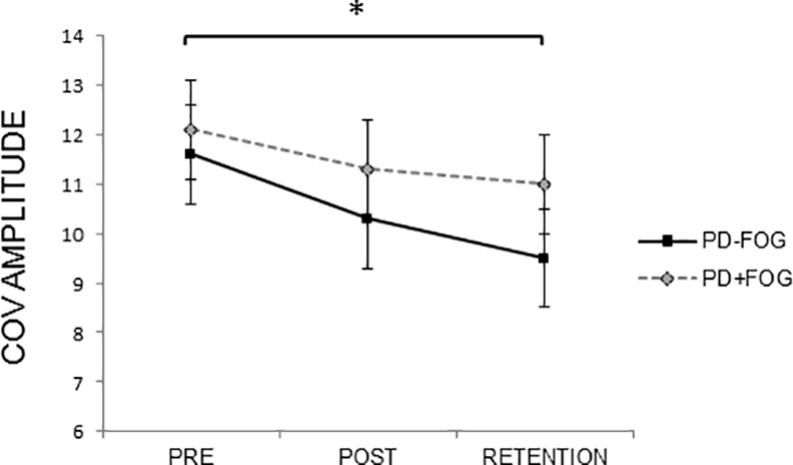
Coefficient of variation of the writing amplitude on the transfer task during the pre, post and retention tests for both groups. Mean and standard errors are presented. For COV_ampl_ a significant main effect of time was found (F = 5.54, p = 0.03), showing improvements in variability of amplitude from pre to retention test in both groups. * indicates significant decrease with p<0.05.

For the transfer task, a main effect of time was found for COV_ampl_ (F = 5.54, p = 0.03). Post hoc tests showed a significant decrease in variability from the pre to the retention test (p = 0.03) in both groups (Fig 4). Furthermore, results showed a significant interaction between Cue and Size (F = 5.38, p = 0.02). In the presence of cues, COV_ampl_ did not differ for writing at large and small size, while, in the absence of cues, COV_ampl_ was significantly larger when writing at large size (p<0.01).

For the automatization task, only a main effect for size was found (F = 4.42, p = 0.04), revealing that COV_ampl_ was significantly smaller during writing at large compared to small size (p = 0.01).

### Accuracy of the Tone-Counting Task

**Fig 5:**
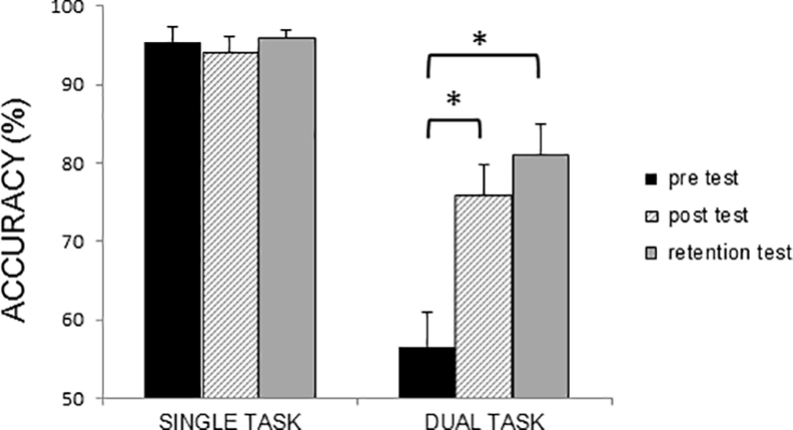
Accuracy of the tone-counting task during the automatization task during single- and dual-task writing. Mean and standard errors are presented. For accuracy of the tone-writing task (%) a significant Time x Task interaction was found (F = 9.17, p<0.01), showing improved performance in dual-task accuracy from the pre to post and pre to retention tests in both groups. * indicates significant increase with p<0.05.

Analyses of accuracy on the tone-counting task, which served as secondary task during the automatization task, showed a significant interaction of Time by Task (F = 9.17, p<0.01). Post hoc tests showed no differences between the three tests for the single task. In contrast, during the dual task, a significant improvement in accuracy was found from pre to post test and from pre to retention test for both groups (p<0.01 for both comparisons) (Fig 5).

## Discussion

The aim of the current study was to investigate the effects of six weeks intensive motor learning of handwriting in patients with PD with and without FOG. In both groups, significant improvements in handwriting were found after completing the training program and this for both single- and dual-task writing. More specifically, significant increases in amplitude, decreases in variability and increases in secondary task accuracy during dual tasking were found from pre- to post-training, underscoring the learning potential in both subgroups of PD. However, although significant short-term improvements were found, retention effects on writing amplitude were only present in the patients without FOG. In patients with FOG, movement amplitude significantly decreased in the six-week follow-up period after the training. Second, the PD+FOG showed a higher cue dependency than PD-FOG during all assessments, showing reduced generalization in the first group. These differences were found irrespective of disease severity differences. Therefore, the present study showed that compared to PD patients without FOG, those with FOG exhibit impaired motor learning, of which retention and generalization of learning are considered hallmarks.

The most prominent finding of this study was the significant lack of consolidation of the primary outcome in the PD+FOG. It has been hypothesized that different modes of learning are involved in the acquisition and consolidation of learning. The initial phase of motor-skill learning has been linked to goal-directed and the later to habitual-based learning [32]. As patients with FOG experience an exaggerated automaticity deficit, they may be forced to rely on the goal-directed mode of action, linked to the early phases of learning, even more so than their non-freezing counterparts [33]. In healthy people, evidence was found that these different learning phases preferentially activate different networks of cortical and subcortical regions [34,35]. Whereas cerebellar mechanisms were mainly associated with adjusting movement kinematics during the early learning phase, the basal ganglia were shown to play an important role when achieving automatization. More specifically, Petzinger et al. [32] related the later phase of learning to increased activation of circuits in the caudal regions of the basal ganglia and the sensorimotor cortex. A recent review by Fasano et al. [36] poses that FOG results from dysfunction within a complex neural circuitry involving multiple brain regions, including several subcortical structures. Shine et al. [37] and Vercruysse et al. [38], for example, showed a significant association between freezing and decreased activation in the basal ganglia. In addition, in a recent DTI study comparing PD-FOG and PD+FOG, white matter alterations in striatofrontal tracts through the putamen, caudate, pallidum and subthalamic nucleus, and in connections of the cerebellar peduncle with subthalamic nucleus and pedunculopontine nucleus were shown in patients with FOG [39]. These functional frontostriatal circuitry impairments may lie at the base of increased difficulties in habitual-based learning in PD+FOG, and as such may explain the lack of retention in this group. It is, at this moment, however, unclear to which extent the neural changes related to gait freezing can be generalized towards the function of the upper limbs and the (re)learning of upper limb movements. Recent studies suggest the existence of a close link between gait freezing and other types of non-gait freezing, including upper limb motor blocks. Similar to gait, motor-cognitive processes seem to interact in the emergence of upper limb freezing, potentially implying a partially overlapping neural circuitry in both phenomena [2]. Future studies are needed to reveal the neural changes underlying different freezing types and their effects on the learning of different types of tasks in patients with and without FOG.

Besides differences in retention of learning, important differences between PD-FOG and PD+FOG were found regarding generalization of learning. The writing of repetitive letters can be considered as a motor task under visual control [40], suggesting that patients with PD could benefit from the provision of visual cues during this type of task. Previous studies on handwriting indeed showed that visual cueing helped to enlarge writing amplitude immediately after training [15,16]. The present study shows for the first time that writing training supported by external cueing also has long-term benefits for PD patients without FOG. Making use of supportive visual information to assist learning, however, runs the risk that patients develop context dependency and experience reduced transfer [41]. Context dependency has been defined as the process by which the environment affects cognitive processing and recall and learning of specific motor skills [32,42]. A specific subtype of context dependency is cue dependency, characterized by significant performance deterioration as soon as the augmented cues used to learn the task are withdrawn. The results of the current study show that PD patients with FOG are particularly prone to develop this type of context dependency. The ability of patients with PD to learn, retain and transfer performance improvements after training has previously been shown to be related to the cognitive demands of the tasks and the cognitive function of the patients [43,44]. Although clear evidence on this matter is still lacking, frontal and prefrontal lobe impairments have been hypothesized to limit rehabilitation efficacy [44]. In support of this view, the lack of transfer towards uncued movement in freezers may be explained by the increased cognitive problems in this PD subgroup [20]. Freezers particularly experience problems with regard to executive functioning [10,12], which was previously found to strongly correlate with grey matter atrophy in the inferior parietal lobule [45]. Furthermore, Tessitore et al. [46] showed that PD+FOG exhibit a significantly reduced functional connectivity within the executive-attention and visual networks. As long as external cues are provided to guide motor performance, freezers are probably able to, at least partly, compensate for these deficits. However, the cognitive reserve in this group seems insufficient to maintain performance levels under higher cognitive loads, i.e. when movement has to be generated internally.

The current results further extend the findings on handwriting training by Nackaerts et al. [9] in PD patients without FOG and can be highly important to develop efficient rehabilitation programs for different subgroups of patients with PD. As both patient groups experienced significant learning effects immediately after training, this points to the therapeutic potential of handwriting training in patients with PD, both with and without FOG. In the rehabilitation of PD+FOG, it seems warranted that cues are provided permanently to ensure that benefits of what was learned persist. The development of wearable technological devices to provide external cueing may be highly useful in this regard [47–49]. Alternatively, it may also be possible that there is an increased necessity for weaning off of cueing in freezers, thereby providing more cognitively challenging learning conditions to stimulate consolidation. However, this approach has to be balanced against the notion that freezers have more cognitive deficits and therefore may reach a cognitive saturation point more quickly during learning. Furthermore, the lack of retention effects in PD+FOG indicates that patients in this group may need more frequent repetition of therapy in order not to lose treatment gains. Nevertheless, the fact that PD patients with FOG were able to improve performance after training, even during dual tasking, is promising as it indicates a potential for neuroplasticity, albeit to a lesser degree than in PD-FOG. A limitation of this study is that only patients in the mid stage of the disease were included, limiting generalization of the results towards the whole PD population. Furthermore, only one type of training was investigated, i.e. handwriting training supported by extensive visual cueing. Future studies are warranted to explore the benefits of different training strategies in patients with and without FOG in different stages of the disease. As well, future work using neuroimaging techniques is needed to shed light on the underlying neural mechanisms of the learning impairments in PD+FOG.

In sum, this study shows that PD patients with FOG, who in general suffer from more severe neural circuitry dysfunction, as well as motor and cognitive impairment, are a group who represent a significant therapeutic challenge. Importantly, significant learning effects were present in this group, both for writing under single- and-dual task conditions. These findings are essential in the context of rehabilitation, as they show that after intensive practice improvements in motor performance can be achieved and dual-task capacity can be increased. However, these gains were not retained after a retention period of six weeks without training in PD+FOG, in contrast to their non-freezing counterparts. Moreover, the improved performance in PD+FOG remained largely dependent on the presence of external cues, showing a lack of generalization towards internally guided movements. It is speculated that these findings can be explained by reduced efficiency of frontostriatal pathways and attentional networks per se in patients with FOG. Further work is required to provide evidence on the causal factors underlying these impairments in motor learning in PD+FOG as well as to extract the factors that may enhance generalization, automatization and retention in this group. A better understanding of the relationship between cognition and motor performance will be crucial in this respect.

## Supporting Information

S1 Dataset Patient characteristics and outcome measures. (XLSX)
